# Using PhenX toolkit measures and other tools to assess urban/rural differences in health behaviors: recruitment methods and outcomes

**DOI:** 10.1186/1756-0500-7-847

**Published:** 2014-11-26

**Authors:** Michael M Hitz, Pat G Conway, Jeanette A Palcher, Catherine A McCarty

**Affiliations:** Essentia Institute of Rural Health, Maildrop: 6AV-2, 502 East Second Street, Duluth, MN 55805 USA

**Keywords:** Design, Epidemiologic research, Questionnaire design, Depression, Health, Occupational, Vacation

## Abstract

**Background:**

The overall study was designed to examine how vacation behavior affects rural and urban Minnesotans and North Dakotans. The purpose of this substudy was to describe the method for sampling, follow-up and response rate by gender and urban/rural location to help inform future studies in this population.

**Methods:**

Essentia health primary care patients (n = 1344) were sent a 21-page self-administered questionnaire. The questionnaire included questions on demographics, work history, perceived stress, work productivity, depression and mania screeners, tobacco use, dietary information, vacation habits, and technology use. Participants were offered $10 to complete the questionnaire.

**Results:**

The overall response to the three mailings to 1344 adults aged 25–64 was 38.8% for a final sample size of 522 completed surveys. Despite the oversampling of males, the total number of responses from males was lower than for females. The response rates between urban and rural locations were nearly identical for the males (33.3% and 33.0% respectively) but higher for rural females than urban females (47.2% and 42.6% respectively). Seventy-eight percent were currently employed. Sixty-nine percent of the participants reported being married, 5.4% were living with a partner, 14% were divorced widowed or separated and 11% were never married. Forty-seven percent of our population had an associate degree or some college, 29% had a Bachelor’s degree or higher, 17% had their diploma or equivalent and 2% had not completed high school.

**Conclusions:**

The goal of the sampling frame and recruitment strategy for this study was to assemble a cohort of approximately 1000 working adults, represented equally by age, gender and rural location. We ended up with a smaller cohort than desired. The law of diminishing returns was observed, although the third mailing was more effective for men than women.

**Electronic supplementary material:**

The online version of this article (doi:10.1186/1756-0500-7-847) contains supplementary material, which is available to authorized users.

## Background

Previously we found a strong inverse association between self-reported frequency of vacations and tension, depression and feelings of exhaustion [1]. We conducted a study to determine if there are urban/rural differences in vacation frequency, to quantify response to mailed questionnaires in a primary care population, and to identify any differential response that may need to be considered in future research with this population. With the need to measure the health of populations served by Accountable Care Organizations (ACO), there will likely be increased need for collecting valid health behavior information by cost effective means.

There is a reasonable body of empirical research related to increasing response rate to surveys. In a pilot study designed to evaluate a mailed version of the usual random digit dialing Behavioral Risk Factor Surveillance Survey in six states across the US, researchers found that response rate to the different methodologies varied by demographic characteristics, that estimated prevalence for sensitive behaviors (such as HIV) were higher in the mailed surveys suggesting better accuracy, and that cell-phone only households could be reached by mail and not by random digit dialing [2]. In a comparison of internet versus mailed questionnaires in an international sample of trauma surgeons, the internet arm had a 13% lower response rate [3]. In addition to lower response rates, internet could be a problem in populations where internet use is low and /or email addresses for the sample population are not known. In a systematic review of mailed questionnaires, the following factors were found to be associated with higher response rates: use of a monetary incentive, shorter questionnaires, personalized letters and questionnaires with colored ink, contacting participants before sending questionnaires, providing a second copy of the questionnaire to non-responders, and questionnaires designed to be of more interest to participants [4]. In this same review, researchers found response rates were lower for sensitive questions and non-university sources. In a mail survey of smoking and respiratory disorders in Norway, researchers found that unemployed, retired individuals and students were less likely to respond [5]. Despite demographic differences in responders and non-responders to a survey of patient perceptions of hospital care, researchers found that increasing participation from 30% to 70% had a modest impact on study conclusions [6].

This paper examines the methods for sampling, follow-up and response rate for a mailed health behaviors questionnaire. We were interested in evaluating the stratified sampling scheme and additional mailings to improve response rate to a behavioral questionnaire.

## Methods

The protocol was reviewed and approved by the Institutional Review Board (IRB) at Essentia Institute of Rural Health. Documentation of written informed consent was waived by the IRB.

A stratified, random sample of adults aged 25 to 64 was selected from Essentia Health family practice patient lists in May 2012. The goal was to have a final sample of 250 urban and 250 rural residents in Minnesota and 250 urban and 250 rural residents in North Dakota. Ideally, most of them would be currently employed. because the overall goal of the study was to examine the association between frequency of vacations, stress and work absenteeism and presenteeism. To achieve gender balance. males were over-sampled by 10% assuming that they would be less likely to respond by that same percentage based on a previous study conducted with a similar population in the upper Midwest [7]. Assuming that 40% of the population would report taking a vacation every two years or less often and that the association of high tension with this frequency of vacation would be similar to what was reported previously (OR = 1.7) [1], 90% power would be available for the urban/rural strata-specific analyses with a significance level of 5%.

The cover letter, signed in blue ink by the Principal Investigator, contained a brief description of the study, the fact that study participation was voluntary, and that participants were offered $10 to complete the survey. The 21-page colored questionnaire was mailed two additional times to non-responders to request their participation. The survey was mailed out on June 20, July 16, and August 13, 2012. Additional file 1.

The questions that were included were selected from the previous study by Dr. McCarty [1], the PhenX Toolkit (http://www.phenxtoolkit.org) [8] that Dr. McCarty used in another study in central Wisconsin, and from the medical literature [9]. The PhenX Toolkit measures are standardized tools assembled by domain experts for use in population-based studies to allow comparison across studies. Table 1 summarizes the items that were included, their source, and the number of questions per item. Some items, such as fruit and vegetable intake and physical activity, were included as indicators of health behavior. The use of standardized tools will allow comparison with other studies.

**Table 1:** **Questions and sources for items included in questionnaire**

Question	PhenX toolkit item number	Reference	Number of items
Frequency of and length of vacations	N/A	[1, 10]	24
Stress-related exhaustion disorder	N/A	[2]	4
Current Age	010101	[8]	1
Gender	010701	[8]	1
Race	010601	[8]	1
Ethnicity	010501	[8]	2
Current marital status	010901	[8]	1
Current educational attainment	011001	[8]	1
Annual family income	011101	[8]	2
Self-reported weight	021502	[8]	1
Maximum adult height	020901	[8]	2
Tobacco – smoking status (adult protocol)	030602	[8]	3
Physical activity screener	150901	[8]	2
Fruits and vegetables intake	050701	[8]	9
Current employment status	011301	[8]	1
Occupational history	060501	[8]	12
Job strain	211201	[8]	15
Perceived stress	180801	[8]	10
Depression – adult	120502	[8]	24
Technology use	N/A	[11]	9
Work productivity and activity impairment instrument	N/A	[9]	6

The data were entered into a Microsoft Access© database. If the subject entered a range, the median was recorded and rounded up to the nearest whole number. If two education boxes were marked, the highest was selected. Non-completed sections were assumed to be refusals to answer. All short answer questions were entered as written on the survey. All data were then checked in a flat field against the hard copy to ensure accuracy. For 13 participants, it appeared that a spouse/partner answered the questionnaire because the gender and birth dates matched information available for the spouse in the Essentia Health medical records. The data from these questionnaires were included in the analyses assuming that the spouse/partner had responded. Where self-reported birth dates did not match Essentia Health records, the self-reported birth date was entered.

SPSS© Version 20.0 was used for the quantitative analyses. A p < 0.05 was considered to be statistically significant.

## Results

**Figure 1:**
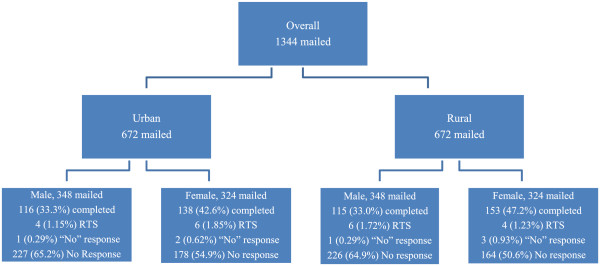
**Survey response by age, gender and location.**

**Figure 2:**
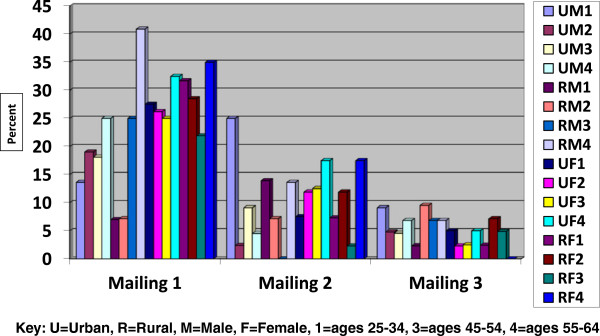
**Percent response to three mailings of self-administered questionnaires by age, gender and location in Minnesota.**

**Figure 3:**
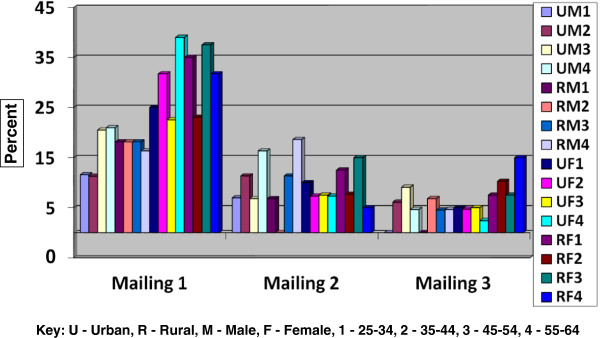
**Percent response to three mailings of self-administered questionnaires by age, gender and location in North Dakota.**

The overall response to the three mailings to 1344 adults aged 25–64 was 38.8%, for a final sample size of 522 completed surveys (Figure 1). Despite the oversampling of males, the total number of responses from males was lower than for females. The response rates between urban and rural locations were nearly identical for the males (33.3% and 33.0% respectively) but higher for rural females than urban females (47.2% and 42.6% respectively).Figures 2 and 3 illustrate the response outcome of the three mailings by state, age, gender and location (urban/rural). The figures illustrate the law of diminishing return, with proportionally fewer of the overall responses occurring with each mailing for most of the strata. The exceptions with the second mailings were the youngest urban and rural males in Minnesota; the overall responses with these groups increased with the second mailing. Generally, the third mailing was more effective for men and women in North Dakota. We found that the third mailing for women in Minnesota was the least effective. In rural Minnesotan men the opposite proved true; having a third mailing was comparatively very productive.

The overall demographics of the sample were as follows: 69% of the participants reported being married and 5.4% were living with partner, 14% were divorced widowed or separated and 11% were never married. In our sample population 2% of people had not completed high school, 17% had graduated high school or received their GED, 47% had attended some college or received their Associates Degree, 23% completed their Bachelors and 9% completed post Bachelor’s degrees. There were minor inconsistencies when the participants were asked about whether they were employed at the time. One-hundred fifteen people reported that they were not working and then responded affirmatively when asked if they had worked more than 35 hours in the past week. These participants were categorized as unemployed. Seventy-seven percent of the participants reported that they were working at the time of the survey.

A comparison of some of the basic demographic information from the stratified sample with US census data reveals that the study sample was more likely to be female, better educated, have higher household incomes (although difficult to compare directly because the questions asked are not identical), and have fewer average number of people per house, with the exception of Jamestown, ND (Table 2). The differences are not statistically significant due to small numbers in the sample strata.

**Table 2:** **Comparison of participant demographics with city-specific US Census data (** http://quickfacts.census.gov **, accessed April 2, 2014)**

Demographic	Duluth census	Duluth study sample	Hibbing census	Non-Duluth study sample	Fargo census	Fargo study sample	Jamestown census	Non-Fargo study sample
Female percent	51.0	53.4	51.6	53.8	49.6	55.3	49.8	62.8
White percent	90.4	97.7	95.9	96.2	90.2	96.7	90.0	99.3
Black or African American percent	2.3	0.8	0.6	0.0	2.7	0.0	0.8	0.0
American Indian	2.5	3.8	0.9	4.6	1.4	0.0	1.8	0.7
Bachelor’s degree or higher	32.0	35.7	15.8	26.3	39.0	44.7	25.4	26.3
Average persons per household	2.22	1.99	2.14	1.83	2.14	1.74	2.15	2.21
Median household income	$41,311	$50,000-$74,999	$37,500	$50,000-$74,999	$44,304	$50,000-$74,999	$45,679	$50,000-$74,999

## Discussion

The goal of the sampling frame and recruitment strategy for this study was to assemble a cohort of approximately 1000 working adults, represented equally by age, gender and urban/rural location. We ended up with a smaller cohort than desired, despite using a recruitment strategy in a similar population recently that resulted in a much higher response rate [12].

There has been substantial research and concern about decreasing participation rates in epidemiologic studies and the potential for non-response bias to impact study conclusions [13, 14]. Women consistently participate at higher rates than men but other potential predictors of response such as age and ethnicity have been inconsistent. Although lower response rates increase the possibility of non-response bias to adversely affect study conclusions, they do not necessarily do so. A research agenda recently released by the National Academy of Sciences listed a number of recommendations for research to help define the problem of non-response, identify why people do take part in surveys, document cost implications of non-response, and identification of plans, policies and procedures to overcome the problem of non-response [15]. We feel that the results of our study contribute to this research agenda and provide guidance for recruiting survey respondents in the upper Midwest.

Reasons that have been suggested for increasing non-participation in studies include the proliferation of research and marketing studies, a general decrease in volunteerism, a growing disillusionment with science, and increased demands on study participants [13]. We do not have information about why people chose not to participate in this survey. In addition to determining the potential bias that may have been introduced by non-response, future research aimed at the identification of barriers to participation and suggestions to overcome those barriers would be useful. We attempted to keep the survey as short as possible so that the length and time commitment would not be a deterrent to participation. Despite being shorter than a similar survey conducted by one of the investigators (CAM) recently [12], the response rate to the current survey was lower (39% versus 70%). A likely reason for this discrepancy is the fact that the earlier questionnaire was mailed to adults who had already participated in a biobank and were therefore predisposed to participate in research studies.

## Conclusions

The results of our recruitment provide lessons for future research in working aged adults in the upper Midwest. Despite the oversampling of males assuming a lower response rate, men responded at an even lower rate than anticipated. For a balanced sample in the future, additional oversampling of men would need to occur or alternative means to reach them, such as on-line surveys, the success of which is dependent on the use of computers and internet access in the intended study population. For women, two survey mailings are probably most cost-effective because we observed the law of diminishing returns with the third mailing. Overall, the third mailing was more useful for men than women.

Few studies have been published that used measures from the PhenX toolkit. We found the tool easy to use, although some modifications were needed to allow for self-administration. With increased used, there will be an ever expanding resource for comparisons.

## Electronic supplementary material

Additional file 1: **Self-administered questionnaire sued in current study.** (DOC 968 KB)

## Abbreviations

PhenX: Consensus measures for Phenotypes and eXposures.
