# Supplementary material for: Using PhenX toolkit measures and other tools to assess urban/rural differences in health behaviors: recruitment methods and outcomes
Source: BMC Res Notes. 2014 Nov 26;7:847. doi: 10.1186/1756-0500-7-847 (PMC4289386; doi:10.1186/1756-0500-7-847)
Supplement: Supplementary file 1 — Additional file 1: Self-administered questionnaire sued in current study. (DOC 968 KB) [file 13104_2013_3440_MOESM1_ESM.doc]

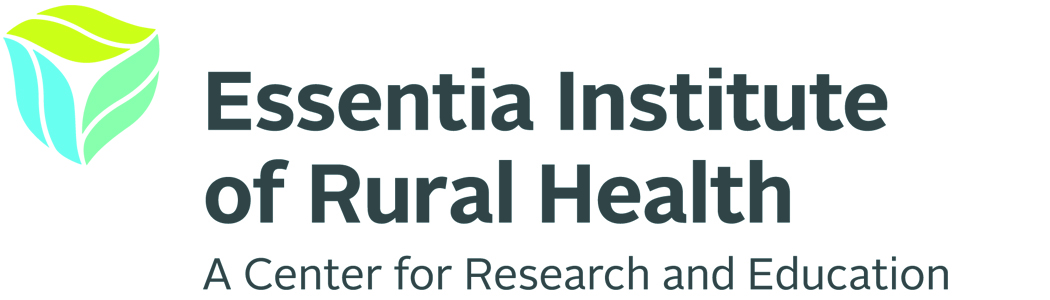


Thank you for taking the time to participate in our study. Please remember that all information that you provide for this survey will be kept strictly confidential.

This questionnaire will provide us information about your demographics, environmental data, mental health, vacation habits, and technology use. We will be using this information to determine if there is any relationship between vacations, depression, and work productivity. Most of the questions on this survey came from a tool kit called PhenX. PhenX takes information that is known to be associated with certain diseases and allows different researchers to use the same questions in their research. Collecting the same data will enable researchers to have access to similar data across a variety of locations.

Please fill out the questions on the following pages to the best of your ability. There will be some questions that will have you skip to another question or section if it does not apply to you.

When you have finished filling out the questionnaire place your questionnaire into the prepaid self addressed envelope and mail it back to us. Once we have received your questionnaire, we will send you a $10.00 dollar check.

**Health Behaviors, Work ,Vacations, and Stress**

**Your completion of this questionnaire will serve as your consent to be in this research study.**

**Demographics**

**1. What is your birth date?** MM/DD/YYYY________________

**2. Are you male or female?**

[ ] MALE

[ ] FEMALE

**3. What race do you consider yourself to be? Please select 1 or more of these categories**.

[ ] WHITE

[ ] BLACK/AFRICAN AMERICAN

[ ] INDIAN (AMERICAN)

[ ] ALASKA NATIVE

[ ] NATIVE HAWAIIAN

[ ] GUAMANIAN

[ ] SAMOAN

[ ] ASIAN INDIAN

[ ] OTHER PACIFIC ISLANDER (SPECIFY)_______________________

[ ] CHINESE

[ ] FILIPINO

[ ] JAPANESE

[ ] KOREAN

[ ] VIETNAMESE

[ ] OTHER ASIAN

(SPECIFY)_______________________

[ ] SOME OTHER RACE (SPECIFY)_______________________

**4. Do you consider yourself Hispanic/Latino?**

[ ] YES [ ] NO (Continue to question 6)

**5. Please check the box of the group that represents your Hispanic origin or ancestry.**

[ ] PUERTO RICAN

[ ] DOMINICAN (REPUBLIC)

[ ] MEXICAN/MEXICANO

[ ] MEXICAN AMERICAN

[ ] CHICANO

[ ] CUBAN

[ ] CUBAN AMERICAN

[ ] CENTRAL OR SOUTH AMERICAN

[ ] OTHER LATIN AMERICAN

[ ] OTHER HISPANIC

1. **Are you now married, widowed, divorced, separated, never married or living with a partner?**

[ ] MARRIED

[ ] WIDOWED

[ ] DIVORCED

[ ] SEPARATED

[ ] NEVER MARRIED

[ ] LIVING WITH PARTNER

**7. How many people, beside yourself, are living or staying at your same address?**Number of people: __ __ (two digit entry)

INCLUDE everyone who is living or staying here for more than 2 months.
INCLUDE anyone else staying here who does not have another place to stay, even if they are here for 2 months or less.
DO NOT INCLUDE anyone who is living somewhere else for more than 2 months, such as a college student living away or someone in the Armed Forces on deployment.

Fill out the following questions for everyone, including you, who is living or staying at this address for more than 2 months.

| Person | What is the Person’s first and last initials? | How is this person related to you? | |
| --- | --- | --- | --- |
| 1 |  | [ ] Husband or wife [ ] Son-in-law or aughter-in-law [ ] Biological son or daughter [ ] Other relative [ ] Adopted son or daughter  [ ] Roomer or boarder  [ ] Stepson or stepdaughter  [ ] Housemate or roommate | [ ] Brother or sister [ ] Unmarried partner  [ ] Father or mother  [ ] Foster child  [ ] Grandchild  [ ] Other nonrelative [ ] Parent-in-law |
| 2 |  | [ ] Husband or wife [ ] Son-in-law or aughter-in-law [ ] Biological son or daughter [ ] Other relative [ ] Adopted son or daughter  [ ] Roomer or boarder  [ ] Stepson or stepdaughter  [ ] Housemate or roommate | [ ] Brother or sister [ ] Unmarried partner  [ ] Father or mother  [ ] Foster child  [ ] Grandchild  [ ] Other nonrelative [ ] Parent-in-law |
| 3 |  | [ ] Husband or wife [ ] Son-in-law or aughter-in-law [ ] Biological son or daughter [ ] Other relative [ ] Adopted son or daughter  [ ] Roomer or boarder  [ ] Stepson or stepdaughter  [ ] Housemate or roommate | [ ] Brother or sister [ ] Unmarried partner  [ ] Father or mother  [ ] Foster child  [ ] Grandchild  [ ] Other nonrelative [ ] Parent-in-law |
| 4 |  | [ ] Husband or wife [ ] Son-in-law or aughter-in-law [ ] Biological son or daughter [ ] Other relative [ ] Adopted son or daughter  [ ] Roomer or boarder  [ ] Stepson or stepdaughter  [ ] Housemate or roommate | [ ] Brother or sister [ ] Unmarried partner  [ ] Father or mother  [ ] Foster child  [ ] Grandchild  [ ] Other nonrelative [ ] Parent-in-law |

**8. What is the total household income?**

[ ] Less than $10,000

[ ] $10,000 – $14,999

[ ] $15,000 – $24,999

[ ] $25,000 – $34,999

[ ] $35,000 – $49,999

[ ] $50,000 – $74,999

[ ] $75,000 – $99,999

[ ] $100,000 – $149,999

[ ] $150,000 – $199,999

[ ] More than $200,000

**9. What is the highest grade or level of school you have completed or the highest degree you have received?**

[ ] NEVER ATTENDED/ KINDERGARTEN ONLY

[ ] 1ST GRADE

[ ] 2ND GRADE

[ ] 3RD GRADE

[ ] 4TH GRADE

[ ] 5TH GRADE

[ ] 6TH GRADE

[ ] 7TH GRADE

[ ] 8TH GRADE

[ ] 9TH GRADE

[ ] 10TH GRADE

[ ] 11TH GRADE

[ ] 12TH GRADE, NO DIPLOMA

[ ] HIGH SCHOOL GRADUATE

[ ] GED OR EQUIVALENT

[ ] SOME COLLEGE, NO DEGREE

[ ] ASSOCIATE DEGREE: OCCUPATIONAL, TECHNICAL, OR VOCATIONAL PROGRAM

[ ] ASSOCIATE DEGREE: ACADEMIC PROGRAM

[ ] BACHELOR'S DEGREE (EXAMPLE: BA, AB, BS, BBA)

[ ] MASTER'S DEGREE (EXAMPLE: MA, MS, MEng, MEd, MBA)

[ ] PROFESSIONAL SCHOOL DEGREE (EXAMPLE: MD, DDS, DVM, JD)

[ ] DOCTORAL DEGREE (EXAMPLE: PhD, EdD)

**10. How much do you weigh without clothes or shoes**? ____________ kg/lbs

**11. If you are currently pregnant, how much did you weigh before your pregnancy?**

__________ kg/lbs

**12. How tall are you without shoes?**

|___|___| ENTER NUMBER OF FEET **AND**

|___|___| ENTER NUMBER OF INCHES

**13.** **What is your current employment status?**

[ ] 1 WORKING NOW

[ ] 2 ONLY TEMPORARILY LAID OFF, SICK LEAVE OR MATERNITY LEAVE

[ ] 3 LOOKING FOR WORK, UNEMPLOYED

[ ] 4 RETIRED

[ ] 5 DISABLED, PERMANENTLY OR TEMPORARILY

[ ] 6 KEEPING HOUSE

[ ] 7 STUDENT

[ ] 8 OTHER (SPECIFY):_____________________

**Current job(s)**

This section will be asking questions on your current and previous jobs. This will help us investigate which jobs or work produces the most stress. It will also provide us with information if there is a certain job that will benefit from time away from work..

**1. How many hours did you work last week at all jobs or businesses?**

ENTER NUMBER OF HOURS: |___|___|___|

**2. Do you usually work 35 hours or more per week in total at all jobs or businesses?**

[ ] YES

[ ] NO

**3. For whom did you work at your main job or business?** (What is the name of the company, business, organization or employer?)
IF MORE THAN 1 JOB, RESPOND FOR **MAIN** JOB.

ENTER NAME OF EMPLOYER:___________________________________

**3.a What kind of business or industry is this?** (For example: a TV or radio station, retail shoe store, state labor department, farm.)

ENTER NAME OF BUSINESS OR INDUSTRY:_________________________________

**3.b What kind of work were you doing?** (For example: farming, mail clerk, computer specialist, machine operator, welder, mechanic.)

ENTER NAME OF OCCUPATION:___________________________________

**3.c What were your most important activities on this job?** (For example: sells cars, keeps account books, operates printing press.)

ENTER NAME OF DUTIES:___________________________________

**4. Which of the following best describes the hours you usually
work at your main job or business?**

[ ] A regular daytime schedule: this is work anytime between 6am and 6pm.

[ ] A regular evening shift: this is work anytime between 2pm and midnight.

[ ] A regular night shift: this is work anytime between 9pm and 8am.

[ ] A rotating shift: a work shift that changes periodically from days to evenings or nights.

[ ] Another schedule

**5. About how long have you worked for your employer as your current occupation?**

ENTER NUMBER (OF DAYS, WEEKS, MONTHS OR YEARS)|___|___|___|

[ ] DAYS

[ ] WEEKS

[ ] MONTHS

[ ] YEARS

**6. At your main job how much paid time off do you receive? ___________** days

**6.a Of this time how much do you use in the average year? ___________** days

**6.b If you do not use all of your paid time off, why not?**___________________________________

____________________________________________________________________________________________________________________________________________________________________

**(Longest Held Job)**

**7. Thinking of all the paid jobs you have ever had, what kind of work were you doing the longest?** (For example, electrical engineer, stock clerk, typist, farmer.)

ENTER OCCUPATION:___________________________________

**8. What kind of business or industry did you work in for the longest period of time?** (For example, a TV or radio station, retail shoe store, state labor department, farm, plastics manufacturer.)

BUSINESS/INDUSTRY:____________________________

**9. What were your most important activities on this job or business?** (For example: sells cars, keeps account books, operates printing press.)

NAME OF DUTIES:________________________________________

**10. About how long did you work at that job or business?**

NUMBER (OF DAYS, WEEKS, MONTHS OR YEARS)|___|___|___|

[ ] DAYS

[ ] WEEKS

[ ] MONTHS

[ ] YEARS

**Job Strain**

**11. This Table will ask you questions to evaluate the stress level that is associated with your job. Please respond to the following statements from 1 (I strongly disagree) to 5 (I strongly agree).**

| My job requires that I learn new things | [ ] 1 [ ] 2 [ ] 3 [ ] 4 [ ] 5 |
| --- | --- |
| My job requires a lot of repetitive work. | [ ] 1 [ ] 2 [ ] 3 [ ] 4 [ ] 5 |
| My job requires me to be creative | [ ] 1 [ ] 2 [ ] 3 [ ] 4 [ ] 5 |
| My job allows me to make a lot of decisions on my own | [ ] 1 [ ] 2 [ ] 3 [ ] 4 [ ] 5 |
| My job requires a high level of skill. | [ ] 1 [ ] 2 [ ] 3 [ ] 4 [ ] 5 |
| On my job, I have very little freedom to decide how I do my work | [ ] 1 [ ] 2 [ ] 3 [ ] 4 [ ] 5 |
| I get to do a variety of different things on my job | [ ] 1 [ ] 2 [ ] 3 [ ] 4 [ ] 5 |
| I have a lot of say about what happens on my job | [ ] 1 [ ] 2 [ ] 3 [ ] 4 [ ] 5 |
| Sometimes people lose jobs they want to keep. How likely is it that during the next couple of years you will lose your present job with your employer? | [ ] 1 [ ] 2 [ ] 3 [ ] 4 [ ] 5 |
| Do you have a problem with exposure to dangerous chemicals on your job? | [ ] 1 [ ] 2 [ ] 3 [ ] 4 [ ] 5 |
| Do you have a problem with exposure to air pollution from dusts, smoke, gas, fumes, fibers, or other things on your job? | [ ] 1 [ ] 2 [ ] 3 [ ] 4 [ ] 5 |
| Do you have a problem with exposure to things placed or stored dangerously on you job? | [ ] 1 [ ] 2 [ ] 3 [ ] 4 [ ] 5 |
| Do you have a problem with exposure to dirty or badly maintained areas at your workplace? | [ ] 1 [ ] 2 [ ] 3 [ ] 4 [ ] 5 |
| Do you have a problem with risk of catching diseases on your job? | [ ] 1 [ ] 2 [ ] 3 [ ] 4 [ ] 5 |
| Do you have a problem with dangerous tools, machinery, or equipment? | [ ] 1 [ ] 2 [ ] 3 [ ] 4 [ ] 5 |
| Do you have exposure to fire, burns, or shocks? | [ ] 1 [ ] 2 [ ] 3 [ ] 4 [ ] 5 |
| Do you have a problem with exposure to dangerous work methods on your job? | [ ] 1 [ ] 2 [ ] 3 [ ] 4 [ ] 5 |
| My supervisor is concerned about the welfare of those under him/her. | [ ] 1 [ ] 2 [ ] 3 [ ] 4 [ ] 5 |
| My supervisor pays attention to what I am saying. | [ ] 1 [ ] 2 [ ] 3 [ ] 4 [ ] 5 |
| My supervisor is helpful in getting the job done. | [ ] 1 [ ] 2 [ ] 3 [ ] 4 [ ] 5 |
| My supervisor is successful in getting people to work together. | [ ] 1 [ ] 2 [ ] 3 [ ] 4 [ ] 5 |
| People I work with are competent in doing their jobs. | [ ] 1 [ ] 2 [ ] 3 [ ] 4 [ ] 5 |
| People I work with take a personal interest in me. | [ ] 1 [ ] 2 [ ] 3 [ ] 4 [ ] 5 |
| People I work with are friendly. | [ ] 1 [ ] 2 [ ] 3 [ ] 4 [ ] 5 |
| People I work with are helpful in getting the job done. | [ ] 1 [ ] 2 [ ] 3 [ ] 4 [ ] 5 |

**12. How satisfied are you with your job?**

[ ] Not at all

[ ] Not too

[ ] Somewhat

[ ] Very

**13. Would you advise a friend to take this job?**

[ ] Advise against

[ ] Have doubts about it

[ ] Strongly recommend

**14. Would you take this job again?**

[ ] Without hesitation

[ ] Have second thoughts

[ ] Definitely not

**15. How likely is it that you will find a new job in the next year?**

[ ] Not at all

[ ] Somewhat

[ ] Very likely

**16 Is this job what you wanted when you applied for it?**

[ ] Not very much like

[ ] Somewhat like

[ ] Very much

**Work Productivity**

The following questions ask about the effect of your health problems on your ability to work and perform regular activities. By health problems we mean any physical or emotional problem or symptom. Please fill in the blanks or circle a number, as indicated.

The next questions are about the **past seven days**, not including today.

**19. During the past seven days, how many hours did you miss from work because of your health problems?** Include hours you missed on sick days, times you went in late, left early, etc., because of your health problems. Do not include time you missed to participate in this study.

*_____*hours

**20. During the past seven days, how many hours did you miss from work because of any other reason, such as vacation, holidays, time off to participate in this study?**

_____hours

**21. During the past seven days, how many hours did you actually work?**

_____hours (If “0”, skip to question 23.)

**22.During the past seven days, how much did your health problems affect your productivity while you were working?**

Think about days you were limited in the amount or kind of work you could do, days you accomplished less than you would like, or days you could not do your work as carefully as usual. If health problems affected your work only a little, choose a low number. Choose a high number if health problems affected your work a great deal*.*

Consider only how much health problems affected
productivity while you were working.

| Health problems had no effect on my work |  |  |  |  |  |  |  |  |  |  |  | Health problems completely prevented me from working |
| --- | --- | --- | --- | --- | --- | --- | --- | --- | --- | --- | --- | --- |
|  | 0 | 1 | 2 | 3 | 4 | 5 | 6 | 7 | 8 | 9 | 10 |  |

CIRCLE A NUMBER

**23. During the past seven days, how much did your health problems affect your ability to do your regular daily activities, other than work at a job?**

By regular activities, we mean the usual activities you do, such as work around the house, shopping, childcare, exercising, studying, etc. Think about times you were limited in the amount or kind of activities you could do and times you accomplished less than you would like. If health problems affected your activities only a little, choose a low number. Choose a high number if health problems affected your activities a great deal.

Consider only how much health problems affected your ability
to do your regular daily activities, other than work at a job.

| Health problems had no effect on my daily activities |  |  |  |  |  |  |  |  |  |  |  | Health problems completely prevented me from doing my daily activities |
| --- | --- | --- | --- | --- | --- | --- | --- | --- | --- | --- | --- | --- |
|  | 0 | 1 | 2 | 3 | 4 | 5 | 6 | 7 | 8 | 9 | 10 |  |

CIRCLE A NUMBER

**24. Do you currently feel, and have felt for more than 2 weeks, physically and/or mentally exhausted?**

[ ] Yes

[ ] No

**25. Do you consider this exhaustion to be caused by long-term stress exposure?**

[ ] Yes

[ ] No

**26.** During the last 2 weeks, have you experienced (answers: Yes or No):

|  | Yes | No |
| --- | --- | --- |
| Concentration or memory problems? | [ ] | [ ] |
| Markedly reduced capacity to tolerate demands or to work under time pressure? | [ ] | [ ] |
| Emotional instability or irritability? | [ ] | [ ] |
| Sleeping problems? | [ ] | [ ] |
| Physical weakness or being more easily fatigued? | [ ] | [ ] |
| Physical symptoms such as muscular pain, chest pain, palpitations, gastrointestinal problems, vertigo or increased sensitivity to sounds? | [ ] | [ ] |

**27. Have the complaints above (questions 24-26) markedly decreased your well-being and/or your functional capacity (work ability, family life, leisure activities or other important ways)?**

[ ] Yes, to a great extent

[ ] Yes, somewhat

[ ] No, not at all

**Perceived Stress**

**28. The questions in this section ask you about your feelings and thoughts during the last month. In each case, you will be asked to indicate by marking how often you felt or thought a certain way on a scale from 1 to 5 (1 Never; 2 Almost Never; 3 Sometimes; 4 Fairly Often; 5 Very Often**).

| In the last month, how often have you been upset because of something that happened unexpectedly? | [ ] 1 [ ] 2 [ ] 3 [ ] 4 [ ] 5 |
| --- | --- |
| In the last month, how often have you felt that you were unable to control the important things in your life? | [ ] 1 [ ] 2 [ ] 3 [ ] 4 [ ] 5 |
| In the last month, how often have you felt nervous and “stressed”? | [ ] 1 [ ] 2 [ ] 3 [ ] 4 [ ] 5 |
| In the last month, how often have you felt confident about your ability to handle your personal problems? | [ ] 1 [ ] 2 [ ] 3 [ ] 4 [ ] 5 |
| In the last month, how often have you felt that things were going your way? | [ ] 1 [ ] 2 [ ] 3 [ ] 4 [ ] 5 |
| In the last month, how often have you found that you could not cope with all the things that you had to do? | [ ] 1 [ ] 2 [ ] 3 [ ] 4 [ ] 5 |
| In the last month, how often have you been able to control irritations in your life? | [ ] 1 [ ] 2 [ ] 3 [ ] 4 [ ] 5 |
| In the last month, how often have you felt that you were on top of things? | [ ] 1 [ ] 2 [ ] 3 [ ] 4 [ ] 5 |
| In the last month, how often have you been angered because of things that were outside of your control? | [ ] 1 [ ] 2 [ ] 3 [ ] 4 [ ] 5 |
| In the last month, how often have you felt difficulties were piling up so high that you could not overcome them | [ ] 1 [ ] 2 [ ] 3 [ ] 4 [ ] 5 |

**29. In general, how satisfied are you with your life? (1 being very dissatisfied to 5 very satisfied)**

| [ ] 1 | [ ] 2 | [ ] 3 | [ ] 4 | [ ]5 |
| --- | --- | --- | --- | --- |

**Tobacco Use**

**1. Have you smoked at least 100 cigarettes in your entire life?**
(100 CIGARETTES = APPROXIMATELY 5 PACKS)

[ ] Yes

[ ] No If Question 1 is "No" proceed to Physical Activity section on the next page.

**2. Do you now smoke cigarettes every day, some days, or not at all?**

[ ] Every day

[ ] Some days

[ ] Not at all

**3. Have you EVER smoked cigarettes EVERY DAY for at least 6 months?**

[ ] Yes

[ ] No

**Physical Activity**

**4. Section I: On-The-Job Activity**

Please check the box next to the one statement that best describes the kinds of physical activity you usually performed whilst on the job this last year. If you are not gainfully employed outside the

home but perform work around the home **regularly**, indicate that activity in this section.

| [ ] A. | If you have no job or regular work, check Box A and go on to Section II. |
| --- | --- |
| [ ] B. | I spent most of the day sitting or standing. When I was at work I did such things as writing, typing, talking on the telephone, assembling small parts or operating a machine that takes very little exertion or strength. If I drove a car or truck while at work, I did not lift or carry anything for more than a few minutes each day. |
| [ ] C. | I spent most of the day walking or using my hands and arms in work that required moderate exertion. When I was at work I did such things as delivering mail, patrolling on guard duty, mechanical work on automobiles or other large machines, house painting or operating a machine that requires some moderate activity. If I drove a truck or lift, my job required me to lift and carry things frequently. |
| [ ] D. | I spent most of the day lifting or carrying heavy objects or moving most of my body in some other way. When I was at work, I did such things as stacking cargo or inventory, handling parts or materials, or I did work like that of a carpenter who builds structures or a gardener who does most of the work without machines. |
| [ ] E. | I spent most of the day doing hard physical labor. When I was at work I did such things as digging or chopping with heavy tools, or carrying heavy loads (bricks, for example) to the place where they are to be used. If I drove a truck or operated equipment, my job also required me to do hard physical work most of the day with only short breaks. |

**5. Section II: Leisure-time Activity**

Please check the box next to the **one** statement which **best** describes the way you spent your leisure-time during most of the last year.

| [ ] F. | Most of my leisure time was spent without very much physical activity. I mostly did things like watching television, reading or playing cards. If I did anything else, it was likely to be light chores around the house or yard, or some easy-going game like bowling or catch. Only once or twice a month did I do anything more vigorous like jogging, playing tennis or active gardening. |
| --- | --- |
| [ ] G. | Weekdays, when I got home from work, I did few active things. But most weekends I was able to get outdoors for some light exercise- going for walks, playing a round of golf (without motorized carts), or doing some active chores around the house. |
| [ ] H. | Three times per week, on the average, I engaged in some moderate activity- such as brisk walking or slow jogging, swimming or riding a bike for 15-20 minutes or more. Or I spent 45 minutes to an hour or more doing moderately difficult chores- such as raking or washing windows, mowing the lawn or vacuuming, or playing games such a doubles tennis or basketball. |
| [ ] I. | During my leisure time over the past year, I engaged in a regular program of physical fitness involving some kind of heavy physical activity at least three times per week. Examples of heavy physical activity are: jogging, running or riding fast on a bicycle for 30 minutes or more; heavy gardening or other chores for an hour or more; active games or sports such as handball or tennis for an hour or more; or a regular program involving calisthenics and jogging or the equivalent for 30 minutes or more. |
| [ ] J. | Over the past year I engaged in a regular program of physical fitness along the lines described n the last paragraph (I), but I did it almost daily- five or more times per week. |

**Dietary Habits**

These questions are about the different kinds of foods you ate or drank during the PAST MONTH, that is, the past 30 days. When answering, please include meals and snacks eaten at home, at work or school, in restaurants, and anyplace else. This information will be used to help determine lifestyles and other contributing factors to stress and depression.

**6. During the past month, how often did you drink 100% FRUIT JUICE, such as orange, mango, apple, and grape juices? Do NOT count fruit drinks.** Do NOT include fruit drinks with added sugar like Kool-aid, Hi-C, lemonade, cranberry cocktail, Gatorade, Tampico, and Sunny Delight.

[ ] Never

[ ] 1-3 times last month

[ ] 1-2 times per week

[ ] 3-4 times per week

[ ] 5-6 times per week

[ ] 1 time per day

[ ] 2 times per day

[ ] 3 times per day

[ ] 4 times per day

[ ] 5 or more times per day

**7. During the past month, how often did you eat FRUIT?** COUNT fresh, frozen, or canned fruit. Do NOT count juices. Include fruits such as but not limited to apples, bananas, applesauce, melon, berries, fruit salad, mangos, papayas, oranges, and grapes.

[ ] Never

[ ] 1-3 times last month

[ ] 1-2 times per week

[ ] 3-4 times per week

[ ] 5-6 times per week

[ ] 1 time per day

[ ] 2 times per day

[ ] 3 times per day

[ ] 4 times per day

[ ] 5 or more times per day

**8. During the past month, how often did you eat a green leafy or lettuce SALAD, with or without other vegetables?** INCLUDE spinach salads

[ ] Never

[ ] 1-3 times last month

[ ] 1-2 times per week

[ ] 3-4 times per week

[ ] 5-6 times per week

[ ] 1 time per day

[ ] 2 times per day

[ ] 3 times per day

[ ] 4 times per day

[ ] 5 or more times per day

**9. During the past month, how often did you eat FRENCH FRIES, home fries, or hash brown potatoes?**

[ ] Never

[ ] 1-3 times last month

[ ] 1-2 times per week

[ ] 3-4 times per week

[ ] 5-6 times per week

[ ] 1 time per day

[ ] 2 times per day

[ ] 3 times per day

[ ] 4 times per day

[ ] 5 or more times per day

**10. During the past month, how often did you eat other WHITE POTATOES? COUNT baked potatoes, boiled potatoes, mashed potatoes and potato salad?** Do NOT include yams or sweet potatoes. INCLUDE red-skinned and Yukon Gold potatoes.

[ ] Never

[ ] 1-3 times last month

[ ] 1-2 times per week

[ ] 3-4 times per week

[ ] 5-6 times per week

[ ] 1 time per day

[ ] 2 times per day

[ ] 3 times per day

[ ] 4 times per day

[ ] 5 or more times per day

**11. During the past month, how often did you eat COOKED DRIED BEANS, such as refried beans, baked beans, bean soup, and pork and beans?** Do NOT include green beans.

[ ] Never

[ ] 1-3 times last month

[ ] 1-2 times per week

[ ] 3-4 times per week

[ ] 5-6 times per week

[ ] 1 time per day

[ ] 2 times per day

[ ] 3 times per day

[ ] 4 times per day

[ ] 5 or more times per day

**12. During the past month, not counting what you just told me about (lettuce salads, white potatoes, cooked dried beans), and not counting rice, how often did you eat OTHER VEGETABLES?** Examples of other vegetables include tomatoes, string beans, carrots, corn, sweet potatoes, cabbage, bean sprouts, collard greens, and broccoli.

[ ] Never

[ ] 1-3 times last month

[ ] 1-2 times per week

[ ] 3-4 times per week

[ ] 5-6 times per week

[ ] 1 time per day

[ ] 2 times per day

[ ] 3 times per day

[ ] 4 times per day

[ ] 5 or more times per day

**13. During the past month, how often did you have TOMATO SAUCES such as spaghetti sauce or pizza with tomato sauce?**

[ ] Never

[ ] 1-3 times last month

[ ] 1-2 times per week

[ ] 3-4 times per week

[ ] 5-6 times per week

[ ] 1 time per day

[ ] 2 times per day

[ ] 3 times per day

[ ] 4 times per day

[ ] 5 or more times per day

**14. During the past month, how often did you have SALSA?**

[ ] Never

[ ] 1-3 times last month

[ ] 1-2 times per week

[ ] 3-4 times per week

[ ] 5-6 times per week

[ ] 1 time per day

[ ] 2 times per day

[ ] 3 times per day

[ ] 4 times per day

[ ] 5 or more times per day

**Depression**

**Part I: Depression Screener from the Composite International Diagnostic Interview - Short Form**

**1a. Have you ever had a time in your life when you felt sad, blue, or depressed for two weeks or more in a row?**

[ ] Yes

[ ] No

**1.b Have you ever had a time in your life lasting two weeks or more when you lost interest in most things like hobbies, work, or activities that usually give you pleasure?**

[ ] Yes

[ ] No

If Yes to either of the two, continue to the next question. If No to both continue to the next portion labeled **Energy, Avtivity, and Mood** on page 17.

Please think of the two-week period in your life when your feelings of depression or loss of interest were worst:

**2. How much of the day did these feeling usually last?**

[ ] All day long

[ ] Most of the day

[ ] About half of the day (continue to the next portion labeled **Energy, Avtivity, and Mood**)

[ ] Less than half of the day (continue to the next portion labeled **Energy, Avtivity, and Mood**)]

**3. Did you feel this way:**

[ ] Every day

[ ] Almost every day

[ ] Less often (continue to the next portion labeled **Energy, Avtivity, and Mood** on page 17)

**4. Did you feel more tired out or low on energy than is usual for you**?

[ ] Yes

[ ] No

**5. Did you gain or lose weight without trying, or did you stay about the same weight?**

[ ] Gained [go to 101a]

[ ] Lost [go to 101b]

[ ] Both gained and lost weight [go to 101c]

[ ] Stayed about the same or on a diet [go to 102]

**101a. About how much weight did you gain?** __________pounds

**101b. About how much weight did you lose?** __________pounds

**101c. About how much weight did you gain and lose?**

Gained __________pounds

Lost __________ pounds

**6. Did you have more trouble falling asleep than you usually do?**

[ ] Yes

[ ] No [go to 8]

**7. How often did that happen?**

[ ] Every night

[ ] Nearly every night

[ ] Less often

**8. Did you have a lot more trouble concentrating than usual?**

[ ] Yes

[ ] No

**9. People sometimes feel down on themselves, no good, or worthless. Did you feel this way?**

[ ] Yes

[ ] No

**10. Do you think a lot about death - either your own, someone else’s, or death in general?**

[ ] Yes

[ ] No

Please again think of the two-week period in your life when your feelings of depression or loss of interest were worst:

**11. About how many weeks altogether did you feel this way? Count the weeks before, during and after the worst two weeks. The total period of depression/loss of interest was:**

__________weeks

**12. How many periods like this did you have in your life, lasting two or more weeks?**

__________periods

**13. About how old were you the FIRST time you had a period of two weeks like this?** (Whether or not you received any help for it.)

__________years of age when you first felt this way

**14. About how old were you the LAST time you had a period of two weeks like this?** (Whether or not you received any help for it.)

__________years of age when the most recent episode happened

**15. Did you ever tell a professional about these problems?** (medical doctor, psychologist, social worker, counselor, nurse, clergy, or other helping professional)

[ ] Yes

[ ] No

**16. Did you take medication or use drugs or alcohol more than once for these problems?**

[ ] Yes

[ ] No

**17. How much did these problems interfere with your life or activities:**

[ ] A lot

[ ] Some

[ ] A little

[ ] Not at all

**Part II: Depression Symptom Assessment from the Quick Inventory of Depressive Symptoms**

Please indicate the one response to each item that best describes you for the past seven days.

**18. Falling Asleep:**

[ ] I never take longer than 30 minutes to fall asleep.

[ ] I take at least 30 minutes to fall asleep, less than half the time.

[ ] I take at least 30 minutes to fall asleep, more than half the time.

[ ] I take more than 60 minutes to fall asleep, more than half the time.

**19. Sleep During the Night:**

[ ] I do not wake up at night.

[ ] I have a restless, light sleep with a few brief awakenings each night.

[ ] I wake up at least once a night, but I go back to sleep easily.

[ ] I awaken more than once a night and stay awake for 20 minutes or more, more than half the time.

**20. Waking Up Too Early:**

[ ] Most of the time, I awaken no more than 30 minutes before I need to get up.

[ ] More than half the time, I awaken more than 30 minutes before I need to get up.

[ ] I almost always awaken at least one hour or so before I need to, but I go back to sleep eventually.

[ ] I awaken at least one hour before I need to, and can’t go back to sleep.

**21. Sleeping Too Much:**

[ ] I sleep no longer than 7-8 hours/night, without napping during the day.

[ ] I sleep no longer than 10 hours in a 24-hour period including naps.

[ ] I sleep no longer than 12 hours in a 24-hour period including naps.

[ ] I sleep longer than 12 hours in a 24-hour period including naps.

**22. Feeling Sad:**

[ ] I do not feel sad

[ ] I feel sad less than half the time.

[ ] I feel sad more than half the time.

[ ] I feel sad nearly all of the time.

**23. Decreased Appetite:**

[ ] There is no change in my usual appetite.

[ ] I eat somewhat less often or lesser amounts of food than usual.

[ ] I eat much less than usual and only with personal effort.

[ ] - I rarely eat within a 24-hour period, and only with extreme personal effort or when others persuade me to eat.

**24. Increased Appetite:**

[ ] There is no change from my usual appetite.

[ ] I feel a need to eat more frequently than usual.

[ ] I regularly eat more often and/or greater amounts of food than usual.

[ ] I feel driven to overeat both at mealtime and between meals.

**25. Decreased Weight (Within the Last Two Weeks):**

[ ] I have not had a change in my weight.

[ ] I feel as if I’ve had a slight weight loss.

[ ] I have lost 2 pounds or more.

[ ] I have lost 5 pounds or more.

**26. Increased Weight (Within the Last Two Weeks):**

[ ] I have not had a change in my weight.

[ ] I feel as if I’ve had a slight weight gain.

[ ] I have gained 2 pounds or more.

[ ] I have gained 5 pounds or more.

**27. Concentration/Decision Making:**

[ ] There is no change in my usual capacity to concentrate or make decisions.

[ ] I occasionally feel indecisive or find that my attention wanders.

[ ] Most of the time, I struggle to focus my attention or to make decisions.

[ ] I cannot concentrate well enough to read or cannot make even minor decisions.

**28. View of Myself:**

[ ] I see myself as equally worthwhile and deserving as other people.

[ ] I am more self-blaming than usual.

[ ] I largely believe that I cause problems for others.

[ ] I think almost constantly about major and minor defects in myself.

**29. Thoughts of Death or Suicide:**

[ ] I do not think of suicide or death.

[ ] I feel that life is empty or wonder if it’s worth living.

[ ] I think of suicide or death several times a week for several minutes.

[ ] I think of suicide or death several times a day in some detail, or I have made specific plans for suicide or have actually tried to take my life.

**30. General Interest:**

[ ] There is no change from usual in how interested I am in other people or activities.

[ ] I notice that I am less interested in people or activities.

[ ] I find I have interest in only one or two of my formerly pursued activities.

[ ] I have virtually no interest in formerly pursued activities.

**31. Energy Level:**

[ ] There is no change in my usual level of energy.

[ ] I get tired more easily than usual.

[ ] I have to make a big effort to start or finish my usual daily activities (for example, shopping, homework, cooking or going to work).

[ ] I really cannot carry out most of my usual daily activities because I just don’t have the energy.

**32. Feeling Slowed Down:**

[ ] I think, speak, and move at my usual rate of speed.

[ ] I find that my thinking is slowed down or my voice sounds dull or flat

[ ] It takes me several seconds to respond to most questions and I’m sure my thinking is slowed.

[ ] I am often unable to respond to questions without extreme effort.

**33. Feeling Restless:**

[ ] I do not feel restless.

[ ] I’m often fidgety, wringing my hands, or need to shift how I am sitting.

[ ] I have impulses to move about and am quite restless.

[ ] At times, I am unable to stay seated and need to pace around.

**Energy, activity and mood**

At different times in their life everyone experiences changes or swings in energy, activity and mood ("highs and lows" or "ups and downs"). The aim of this questionnaire is to assess the characteristics of the "high" periods.

**1. How are you feeling today compared to your usual state:**

(*Please mark only ONE of the following*)

         [ ] Much worse than usual

         [ ] Worse than usual

         [ ] A little worse than usual

         [ ] Neither better nor worse than usual

         [ ] A little better than usual

         [ ] Better than usual

         [ ] Much better than usual

**2. How are you usually compared to other people?**

Independently of how you feel today, please tell us how you are normally compared to other people, by marking which of the following statements describes you best.

Compared to other people my level of activity, energy and mood...

(*Please mark only ONE of the following*)

         [ ] ... is always rather stable and even

         [ ] ... is generally higher

         [ ] ... is generally lower

         [ ] ... repeatedly shows periods of ups and downs

**3. Please try to remember a period when you were in a "high" state.**

How did you feel then? Please answer all these statements independently of your present condition. Check the box that corresponds with your answer (if it is different from normal).

| In such a state: | Yes | No |
| --- | --- | --- |
| I need less sleep | [ ] | [ ] |
| I feel more energetic and more active | [ ] | [ ] |
| I am more self-confident | [ ] | [ ] |
| I enjoy my work more | [ ] | [ ] |
| I am more sociable (make more phone calls, go out more) | [ ] | [ ] |
| I want to travel and/or do travel more | [ ] | [ ] |
| I tend to drive faster or take more risks when driving | [ ] | [ ] |
| I spend more money/too much money | [ ] | [ ] |
| I take more risks in my daily life (in my work and/or other activities) | [ ] | [ ] |
| I am physically more active (sport etc.) | [ ] | [ ] |
| I plan more activities or projects | [ ] | [ ] |
| I have more ideas, I am more creative | [ ] | [ ] |
| I am less shy or inhibited | [ ] | [ ] |
| I wear more colourful and more extravagant clothes/make-up | [ ] | [ ] |
| I want to meet or actually do meet more people | [ ] | [ ] |
| I am more interested in sex, and/or have increased sexual desire | [ ] | [ ] |
| I am more flirtatious and/or am more sexually active | [ ] | [ ] |
| I talk more | [ ] | [ ] |
| I think faster | [ ] | [ ] |
| I make more jokes or puns when I am talking | [ ] | [ ] |
| I am more easily distracted | [ ] | [ ] |
|  | Yes | No |
| I engage in lots of new things | [ ] | [ ] |
| My thoughts jump from topic to topic | [ ] | [ ] |
| I do things more quickly and/or more easily | [ ] | [ ] |
| I am more impatient and/or get irritable more easily | [ ] | [ ] |
| I can be exhausting or irritating for others | [ ] | [ ] |
| I get into more quarrels | [ ] | [ ] |
| My mood is higher, more optimistic | [ ] | [ ] |
| I drink more coffee | [ ] | [ ] |
| I smoke more cigarettes | [ ] | [ ] |
| I drink more alcohol | [ ] | [ ] |
| I take more drugs (sedatives, anxiolytics, stimulants...) | [ ] | [ ] |

**4. Impact of your "highs" on various aspects of your life**:

|  | Negative | Positive and Negative | Positive | No impact |
| --- | --- | --- | --- | --- |
| Family life |  |  |  |  |
| Social life |  |  |  |  |
| Work |  |  |  |  |
| Leisure |  |  |  |  |

**5. How did people close to you react to or comment on your "highs"?**

(*Please mark ONE of the following*)

[ ] Positively (encouraging or supportive)

[ ] Positively and negatively

[ ] Neutral

[ ] Negatively (concerned, annoyed, irritated, critical)

**6. Length of your "highs" as a rule (on the average):**

(*Please mark ONE of the following*)

[ ] 1 day

[ ] 2-3 days

[ ] 4-7 days

[ ] longer than 1 week

[ ] longer than 1 month

**7. Have you experienced such "highs" in the past twelve months**?

[ ] Yes

[ ] No

**8. If yes, please estimate how many days you spent in "highs" during the last twelve months:**

__________days.

**Frequency and Length of Vacations**

This section is designed to help us understand what is considered a vacation. We will link this data to stress and depression levels. In doing so, we hope to find relationships between vacations and stress and depression.

**1. Please describe your ideal vacation: ________________________________________________**

**____________________________________________________________________________________________________________________________________________________________________________________________________________________________________________________________**

**2. On average, how often have you had a vacation?** Please mark one answer.

[ ] Twice or more per year

[ ] Once a year

[ ] Once every 2-5 years

[ ] Once every 6 years or less

**3. The table below is a chart for the vacations that you have taken over the past year. Please fill in the table in regards to any vacations you have taken from June 2011 to June of 2012 with information to the best of your knowledge.**

| Approximate date of vacation: | How long was your vacation? | Where did you go on vacation? | While on vacation how many hours were spent on work related activities? | During your vacation approximately percent of your time was spent doing the following types of activities: physical activities (hiking, swimming, skiing, etc), social activities (bar hopping, attending concerts, sight seeing, etc), or passive activities (reading a book, watching TV)? | On a scale from 1-10 (with 10 being the best) how much did you enjoy your vacation? |
| --- | --- | --- | --- | --- | --- |
| _________  (MM/YY) | ________ day(s) |  |  | Physical: _______%  Social: _______%  Passive: _______% |  |
| _________  (MM/YY) | ________ day(s) |  |  | Physical: _______%  Social: _______%  Passive: _______% |  |
| _________  (MM/YY) | _______ day(s) |  |  | Physical: _______%  Social: _______%  Passive: _______% |  |
| _________  (MM/YY) | ________ day(s) |  |  | Physical: _______%  Social: _______%  Passive: _______% |  |

**4.** For the following table please respond to the statements on a scale from 1 (I do not agree at all) to 5 (I fully agree) in regards to the way your time is spent while on vacation.

| I forget about work. | [ ]1 [ ]2 [ ]3 [ ]4 [ ]5 |
| --- | --- |
| I don’t think about work at all. | [ ]1 [ ]2 [ ]3 [ ]4 [ ]5 |
| I distance myself from work. | [ ]1 [ ]2 [ ]3 [ ]4 [ ]5 |
| I get a break from the demands of work. | [ ]1 [ ]2 [ ]3 [ ]4 [ ]5 |
| I kick back and relax. | [ ]1 [ ]2 [ ]3 [ ]4 [ ]5 |
| I do relaxing things. | [ ]1 [ ]2 [ ]3 [ ]4 [ ]5 |
| I use the time to relax. | [ ]1 [ ]2 [ ]3 [ ]4 [ ]5 |
| I take time for leisure. | [ ]1 [ ]2 [ ]3 [ ]4 [ ]5 |
| I learn new things. | [ ]1 [ ]2 [ ]3 [ ]4 [ ]5 |
| I seek out intellectual challenges. | [ ]1 [ ]2 [ ]3 [ ]4 [ ]5 |
| I do things that challenge me. | [ ]1 [ ]2 [ ]3 [ ]4 [ ]5 |
| I do something to broaden my horizons. | [ ]1 [ ]2 [ ]3 [ ]4 [ ]5 |
| I feel like I can decide for myself what to do. | [ ]1 [ ]2 [ ]3 [ ]4 [ ]5 |
| I decide my own schedule. | [ ]1 [ ]2 [ ]3 [ ]4 [ ]5 |
| I determine for myself how I will spend my time. | [ ]1 [ ]2 [ ]3 [ ]4 [ ]5 |
| I take care of things the way that I want them done. | [ ]1 [ ]2 [ ]3 [ ]4 [ ]5 |

**Use of Technology**

**1. In the last 12 months, have you used the Internet for personal or work reasons?**

[ ] Yes If yes, how long have you been using the internet? _________years

[ ] No If no please skip to question

**1.a Do you access the internet from:** (Please mark all that apply)

[ ] Home

[ ] Work

[ ] School

[ ] Wireless laptop

[ ] Café

[ ] Public library

[ ] A wireless hand-held device

[ ] Other: (Please Specify)______________________________________________

**1.b How often do you go online during a typical week?**

[ ] Several times a day

[ ] Once a day

[ ] 1-2 days a week

[ ] 3-4 days a week

[ ] 5 days a week

[ ] 6 days a week

**2. Which of the following equipment/services do you have available to you?** (Mark all that apply)

[ ] Cellular phone with internet access

[ ] Cellphone with camera

[ ] Cellphone

[ ] Digital camera

[ ] Pager

[ ] Laptop computer

[ ] Laptop computer with wireless access

[ ] Desktop computer

[ ] Global Positioning System/GPS in vehicle

[ ] Personal Digital Assistant (Palm Pilot, Blackberry)

**3. While on vacation which of the following equipment/services do you usually have available to you?**

[ ] Cellular phone with internet access

[ ] Cellphone with camera

[ ] Cellphone

[ ] Digital camera

[ ] Pager

[ ] Laptop computer

[ ] Laptop computer with wireless access

[ ] Desktop computer

[ ] Global Positioning System/GPS in vehicle

[ ] Personal Digital Assistant (Palm Pilot, Blackberry)

**4. Based on the types of technology listed in the previous question, respond to the following statements on a scale of 1 (low) to 7 (high)**

| I consider my use of technology as: | 1 2 3 4 5 6 7 |
| --- | --- |
| I consider my use of the Internet as: | 1 2 3 4 5 6 7 |
| Compared to my friends, my ownership of technology is: | 1 2 3 4 5 6 7 |

**5. Please share any comments or stories about whether you think technology is making your vacations better or worse.**

**____________________________________________________________________________________________________________________________________________________________________________________________________________________________________________________________**

**Thank you for your time!**
